# Breathing mode assessment with thermography: a pilot study

**DOI:** 10.1590/2317-1782/20232022323en

**Published:** 2024-04-15

**Authors:** Yasmim Carvalho Telson, Renata Maria Moreira Moraes Furlan, Rafael Augusto Magalhães Ferreira, Matheus Pereira Porto, Andréa Rodrigues Motta

**Affiliations:** 1 Programa de Pós-graduação em Ciências Fonoaudiológicas, Faculdade de Medicina, Universidade Federal de Minas Gerais - UFMG - Belo Horizonte (MG), Brasil.; 2 Departamento de Fonoaudiologia, Universidade Federal de Minas Gerais - UFMG - Belo Horizonte (MG), Brasil.; 3 Departamento de Engenharia Mecânica, Universidade Federal de Minas Gerais - UFMG - Belo Horizonte (MG), Brasil.

**Keywords:** Thermography, Speech Language and Hearing Sciences, Respiration, Mouth Breathing, Nose, Mouth

## Abstract

**Purpose:**

To present a method for analyzing breathing modes with infrared thermography.

**Methods:**

This exploratory cross-sectional study used 38 thermal images of inspiration and expiration with nasal breathing and simulated mouth breathing in four nasal breathers without respiratory complaints. Three different data selection forms (line, rectangle, and ellipse) were used to extract the minimum, mean, and maximum temperatures of the regions of interest (nose and mouth) using the FLIR Tools^®^ software.

**Results:**

Among the three selection forms, there was greater temperature variability obtained with the line, revealing limitations in this measurement. There were no differences between the rectangle and ellipse values, showing that both selection forms present similar temperature extraction results. The comparison results between nose and mouth temperatures during inspiration and expiration indicated a statistically significant difference between all measurements, except for mean inspiration temperatures with the rectangle and ellipse. The breathing mode can be distinguished in both inspiration and expiration when using mean mouth temperatures with the rectangle and ellipse.

**Conclusion:**

Breathing modes should be assessed based on mean mouth temperatures during inspiration, using the ellipse.

## INTRODUCTION

Breathing is a physiological function of great relevance for maintaining life^(1)^. The breathing mode is an important factor influencing the balance of structures and other orofacial functions, which is why it demands attention from speech-language-hearing pathologists^(2)^. Habitual mouth breathing can significantly change myofunctional balance, causing, for instance, craniofacial and occlusal deformations, loss of orofacial muscle strength, functional changes in chewing, swallowing, and speech articulation, and changes and adaptations in the body^(2)^.

A limitation of myofunctional assessment is the scarcity of quantitative and precise techniques for detecting functional and/or structural abnormalities that may interfere with the breathing mode. Breathing assessment is restricted to a subjective analysis by the evaluator, as there is no gold standard equipment to be used^(3)^.

Infrared thermography is a non-contact method that evaluates a range of temperatures in a given scene^(4)^. It is a non-invasive, non-radioactive technique capable of capturing the range of infrared radiation emitted by a body or object and converting it into temperature information^(5)^.

Thermal cameras have sensors that respond to a range of the electromagnetic spectrum not perceived by the naked eye (wavelength between 0.75 and 1000 μm)^(6)^. They are responsible for transforming this information into electrical signals that are processed and converted into a visible image, represented in different color scales for the various temperature levels captured^(4)^. The radiation emitted by the body generally depends not only on its temperature but also on emissivity, reflectivity, and transmissivity^(7)^. Therefore, the thermal camera indirectly records the temperature of a body, deducing it through the amount of radiation captured by the lenses.

Thermography has proven to be an important tool in investigating respiratory function. Promising studies have been based on the premise that the temperature around the nostrils fluctuates throughout the respiratory cycle^(8-19)^. During inspiration, cold air from the environment is inhaled, while during expiration, warm air from the lungs is exhaled^(9-11)^. Thermograms are then capable of identifying such changes and representing qualitatively and quantitatively the temperature variation around the nasal vestibule.

The growing interest in thermography to assess breathing arises from the fact that respiratory rate is considered an important vital sign^(9,10)^. Studies also use the technique to detect obstructive sleep apnea during polysomnography^(12)^ and even evaluate breathing in cases of nasal septum perforations^(13)^. As this technique does not require contact, it has also been used to assess children^(14)^ and infants in neonatal units^(15,16)^. In addition to information on temperature changes in the nostrils, studies have used thermography to analyze variations around the mouth, obtaining robust results^(17-19)^.

Given the current use of thermography to assess respiratory function - a focus of interest for speech-language-hearing pathologists in oral motor therapy -, it would be relevant to integrate this instrument in breathing mode assessment. Thus, this study aimed to present a method of analyzing breathing mode using infrared thermography.

## METHODS

This is an exploratory cross-sectional study. Data collection only began after approval by the Institution's Research Ethics Committee under evaluation report number 4.364.887. All participants signed an informed consent form.

The participant inclusion criteria were being 18 years old or over; being a nasal breather; and not having any physical, neurological, and/or cognitive changes that might prevent or hinder collaboration during the examination. The exclusion criteria were using a bandage on the skin and/or other factors that prevented its exposure to a balanced temperature environment; doing physical exercises, acupuncture, massages; having undergone electrical stimulation; having attended saunas or been exposed to the sun for a long time up to 24 hours before taking measurements; using bronchodilators and vasoactive medications; having a fever, allergic rhinitis, flu-like symptoms, and/or a cold on the day of the tests; having orofacial pain; having a beard (men); and being in the fertile or menstrual period (women).

Based on these criteria, four female volunteers were selected by convenience. They were self-reported nasal breathers without respiratory complaints, with a mean age of 33.5 years. All four were speech-language-hearing pathologists working in oral motor therapy; therefore, their report of habitual nasal breathing was reliable. Altogether, the sample included 38 thermograms of all participants, 19 of which with habitual nasal breathing and 19 with simulated mouth/oronasal breathing.

Even though they had knowledge about mouth breathing, the classification of the participants' breathing mode was confirmed in two other stages. Thus, after being asked about their breathing mode (the answer could be nasal, mouth, or oronasal), the participants were assessed to verify whether they could close their lips for at least 2 minutes. To be considered a possible nasal breather, the participant must be able to maintain the lips closed without tension. Lastly, their orofacial muscle tone was assessed, which had to be adequate for them to be classified as a possible nasal breather.

Participants remained in the room for approximately 20 minutes before taking measurements to stabilize the temperature, complying with the recommendations of the American Academy of Thermology^(20)^.

A FLIR SC660 thermal camera (FLIR Inc., Santa Barbara, CA) and a 24-degree FLIR lens (38 mm) were used for the tests. The FLIR SC660 camera was selected because it has good resolution (0.1 °C) and sensitivity (0.03 °C), as well as the image sequence recording tool with determined frequencies, for being able to record radiometric information (temperature values). The acquisition frequency was set at five frames per second. The lens was selected following the best FOV (field of view) criteria for the scene, positioning the camera approximately 1 meter away from the volunteer's face, and maximizing the IFOV (instantaneous field of view) in relation to the main targets (nose and mouth). Thus, temperature changes could be observed throughout the breathing process.

Measurements were taken in a room whose temperature was monitored and maintained at around 20±1 °C^(20)^. From a metrological standpoint, the room temperature must be well controlled to allow for result repeatability and create a basic thermal reference between different volunteers^(4)^.

Regarding the experimental setup of the tests, the thermal imager was stabilized on a tripod to avoid vibration during the experiments. A standard 30° measuring angle was used to visualize the nostrils and mouth during collection better. The participant was then seated on a fixed armless chair with its back against the wall, their feet flat on the floor, approximately 1 meter away from the camera lens.

**Figure 1:**
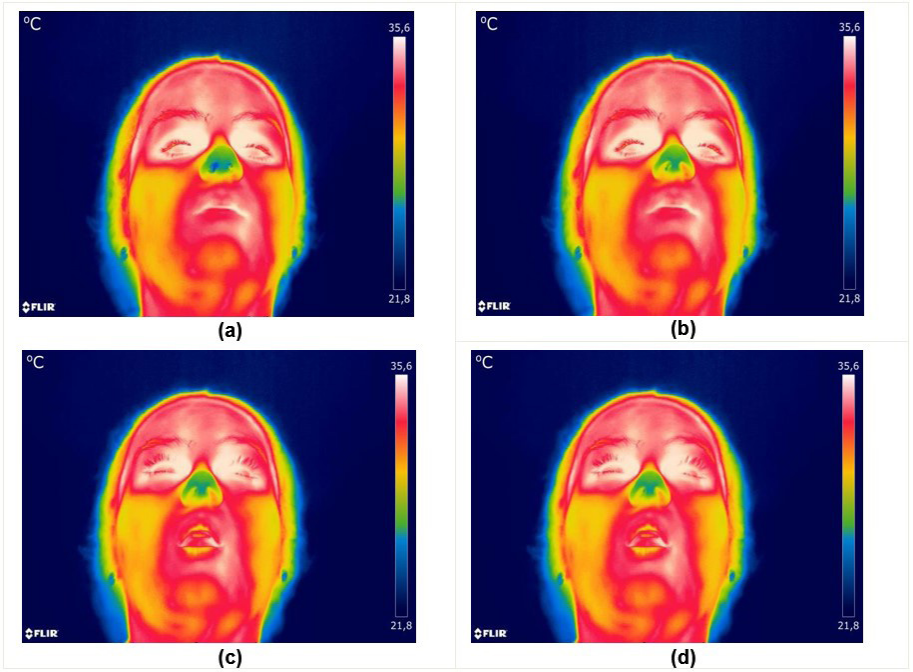
Thermograms selected with the experimental device during the inhaling and exhaling process through the nose (a)-(b) and mouth (c)-(d)

Data were collected in two stages. Initially, the volunteers were filmed breathing normally through the nose for 2 minutes. In the second stage, the volunteers were recorded again for 2 minutes, simulating mouth/oronasal breathing. Figure 1 synthesizes these processes.

FLIR Tools^®^ software was used for the quantitative and qualitative analyses of the image sequences. The emissivity level was set at 0.98, which corresponds to that of the human skin^(21)^.

**Figure 2:**
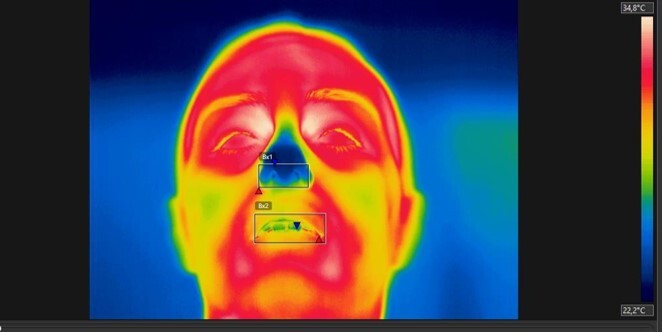
ROIs manually selected through a rectangle in the FLIR Tools software

**Figure 3:**
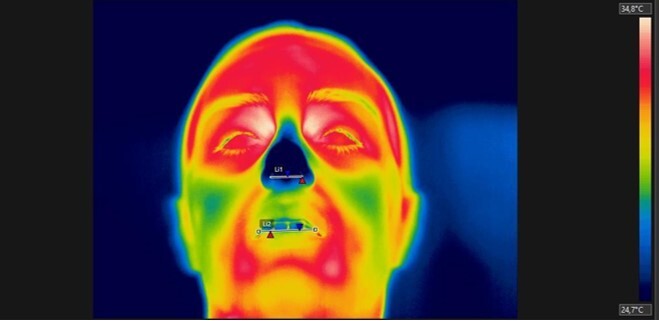
ROIs manually selected through a horizontal line in the FLIR Tools software

**Figure 4:**
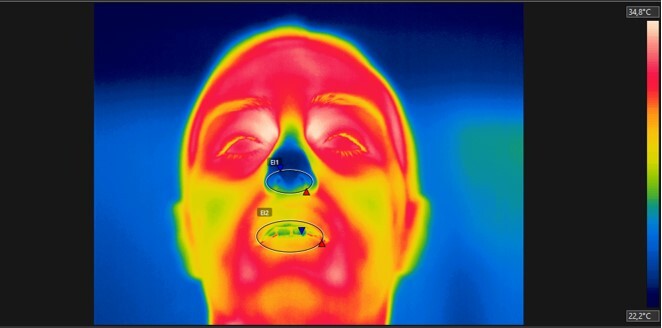
ROIs manually selected through an ellipse in the FLIR Tools software

Three different selection forms - line, ellipse, and rectangle - were used to measure the temperature variation around the nostrils and mouth during inspiration. Firstly, measurements were taken by positioning horizontal lines between the corners of the mouth and between the nostrils. Then, the thermograms were analyzed using the areas of a rectangle and an ellipse, positioned between the corners of the mouth, the cupid's bow, and the lower limit of the lower lip and chin to obtain thermal values of the mouth. As for nose temperatures, another ellipse and another rectangle were placed around the nostrils, the tip of the nose, and the nasolabial angle. Figures 2, 3 and 4 depict these mechanisms.

When the region of interest (ROI) is selected, the FLIR Tools® program presents three values - the maximum, minimum, and mean temperatures of the selected area.

The data were analyzed in IBM SPSS Statistics, version 23. The distribution of continuous variables was tested with the Shapiro-Wilk test, which indicated an asymmetric distribution. Comparisons between the nasal and mouth/oronasal breathing modes and between nose and mouth temperatures were analyzed with the Mann-Whitney test. Comparisons between the minimum, mean, and maximum temperatures were analyzed with the Kruskal-Wallis test. The significance level was set at 5%.

## RESULTS

The temperatures obtained during inspiration and expiration with nasal and mouth/oronasal breathing are compared in Table 1. It shows the following measurements with significant differences in inspiration: mean nose temperature with the ellipse and the rectangle; minimum mouth temperature with the line; and mean mouth temperature with the ellipse, the rectangle, and the line. During expiration, the following ones had statistical differences: mean nose temperature with the line; maximum nose temperature with the line; and mean mouth temperature with the ellipse and rectangle.

**Table 1:** Comparison of temperatures (°C) of the regions of interest during nasal and mouth/oronasal breathing

Variables				Inhaling				Exhaling			
ROI	**Temperature Analyzed**	**Selection form**	**Breathing mode**	**Mean**	**SD**	**VC**	**p-value**	**Mean**	**SD**	**VC**	**p-value**
Nose	Minimum	Ellipse	Nasal	22.0	1.3	6.1	.990	23.8	0.9	3.8	.584
			Mouth	22.0	1.2	5.3		23.7	0.8	3.3	
		Rectangle	Nasal	22.0	1.3	6.0	.939	23.9	0.9	3.8	.750
			Mouth	22.0	1.2	5.3		23.8	0.8	3.4	
		Line	Nasal	22.5	1.9	8.3	.603	24.5	0.8	3.3	.289
			Mouth	22.2	1.4	6.2		24.2	0.8	3.2	
	Mean	Ellipse	Nasal	25.4	1.0	3.9	**.000**	26.5	0.7	2.6	.197
			Mouth	30.8	0.7	2.3		26.2	0.7	2.6	
		Rectangle	Nasal	25.0	1.0	4.0	**.000**	26.4	0.7	2.7	.157
			Mouth	30.9	0.6	1.9		26.0	0.7	2.6	
		Line	Nasal	24.1	1.2	5.2	.269	26.5	0.6	2.1	**.011**
			Mouth	24.5	1.1	4.5		26.0	0.8	3.0	
	Maximum	Ellipse	Nasal	29.3	1.3	4.5	.932	29.5	0.7	2.4	.140
			Mouth	29.3	2.0	6.9		29.0	1.1	3.9	
		Rectangle	Nasal	29.1	0.9	3.2	.428	29.6	0.7	2.3	.076
			Mouth	29.5	1.7	5.7		29.1	1.0	3.3	
		Line	Nasal	25.7	1.2	4.8	.324	29.2	0.6	1.9	**.001**
			Mouth	26.2	1.3	5.0		27.7	1.8	6.6	
Mouth	Minimum	Ellipse	Nasal	27.3	1.4	5.0	.962	29.1	1.1	3.9	.954
			Mouth	27.3	1.3	4.8		29.1	1.1	3.8	
		Rectangle	Nasal	27.2	1.5	5.4	.835	29.3	0.9	3.1	.930
			Mouth	27.2	1.3	4.8		29.3	1.0	3.2	
		Line	Nasal	30.0	0.8	2.8	**.000**	30.0	0.6	2.1	.089
			Mouth	28.3	1.5	5.4		29.2	1.9	6.6	
	Mean	Ellipse	Nasal	25.3	1.5	5.9	**.000**	30.8	0.7	2.2	**.006**
			Mouth	29.9	0.8	2.7		30.1	0.7	2.5	
		Rectangle	Nasal	25.6	1.5	6.0	**.000**	30.9	0.7	2.1	**.003**
			Mouth	30.2	0.7	2.4		30.2	0.8	2.6	
		Line	Nasal	30.9	1.0	3.1	**.000**	30.9	0.6	2.1	.363
			Mouth	29.5	1.1	3.6		30.6	1.5	5.0	
	Maximum	Ellipse	Nasal	33.0	0.7	2.0	.224	32.9	0.8	2.3	.687
			Mouth	32.7	1.0	3.0		33.0	1.0	3.0	
		Rectangle	Nasal	33.0	0.4	1.3	.851	32.9	0.8	2.4	.821
			Mouth	32.9	1.0	3.1		32.8	0.9	2.8	
		Line	Nasal	32.3	1.1	3.5	.124	32.4	1.0	3.0	.496
			Mouth	31.8	0.7	2.1		32.6	0.8	2.5	

**Caption:** p = significance probability (Mann-Whitney test); ROI = region of interest; SD = standard deviation; VC = variation coefficient

The comparison of temperatures according to the selection form during both inspiration and expiration (Table 2) shows differences between the line, rectangle, and ellipse in almost all measurements. This difference was predominant between the line and ellipse and between the line and rectangle - most of the time the line measurements were smaller than the rectangle and ellipse measurements.

**Table 2:** Comparison of temperatures (°C) obtained with the different selection forms: line, ellipse, and rectangle

Variables				Inhaling				Exhaling			
ROI	Breathing Mode	**Temperature**	**Form seleção**	Mean**	**SD**	**VC**	p-value*	Mean**	**SD**	**VC**	p-value*
Nose	Nasal breathing	Minimum	Ellipse	22.0 ^A^	1.3	6.1	**.000**	23.8 ^A^	1.3	5.6	**.000**
			Rectangle	22.0^A^	1.3	6.0		23.9 ^A^	1.3	5.6	
			Line	22.5 ^B^	1.9	8.3		24.5 ^B^	1.8	7.6	
		Mean	Ellipse	25.5 ^A^	1.0	3.9	**.000**	26.5 ^A^	1.0	3.8	**.037**
			Rectangle	25.0^B^	1.0	4.0		26.4 ^A^	1.0	3.8	
			Line	24.0 ^C^	1.2	5.2		26.5 ^A^	1.2	4.7	
		Maximum	Ellipse	29.3 ^A^	1.3	4.5	**.000**	29.5 ^A^	1.3	4.5	**.000**
			Rectangle	29.1 ^A^	0.9	3.2		29.6 ^A^	0.9	3.1	
			Line	25.7 ^B^	1.2	4.8		29.2 ^B^	1.2	4.2	
	Mouth breathing	Minimum	Ellipse	22.0 ^A^	1.2	5.3	**.000**	23.7 ^A^	1.2	5.0	**.000**
			Rectangle	22.0 ^A^	1.2	5.3		23.8 ^A^	1.2	4.9	
			Line	22.2 ^B^	1.4	6.2		24.2 ^B^	1.4	5.7	
		Mean	Ellipse	30.8 ^A^	0.7	2.3	**.000**	26.2 ^A^	0.7	2.7	**.**476
			Rectangle	30.9 ^A^	0.6	1.9		26.0 ^A^	0.6	2.3	
			Line	24.5 ^B^	1.1	4.5		26.0 ^A^	1.1	4.2	
		Maximum	Ellipse	29.3 ^A^	2.0	6.9	**.000**	29.0 ^A^	2.0	6.9	**.000**
			Rectangle	29.5 ^A^	1.7	5.7		29.0 ^A^	1.7	5.8	
			Line	26.2 ^B^	1.3	5.0		27.7 ^B^	1.3	4.7	
Mouth	Nasal breathing	Minimum	Ellipse	27.3 ^A^	1.4	5.0	**.000**	29.1 ^A^	1.4	4.7	**.026**
			Rectangle	27.2 ^A^	1.5	5.4		29.3 ^AB^	1.5	5.0	
			Line	30.0 ^B^	0.8	2.8		30.0 ^C^	0.8	2.8	
		Mean	Ellipse	25.3 ^A^	1.5	5.9	**.000**	30.8 ^A^	1.5	4.8	**.568**
			Rectangle	25.6 ^A^	1.5	6.0		30.9 ^A^	1.5	5.0	
			Line	30.9 ^B^	1.0	3.1		30.9 ^A^	1.0	3.1	
		Maximum	Ellipse	33.0 ^A^	0.7	2.0	**.000**	32.9 ^A^	0.7	2.0	**.000**
			Rectangle	33.0 ^A^	0.4	1.3		32.9 ^A^	0.4	1.3	
			Line	32.3 ^B^	1.1	3.5		32.4 ^B^	1.1	3.5	
	Mouth breathing	Minimum	Ellipse	27.3 ^A^	1.3	4.8	.191	29.0 ^A^	1.3	4.5	.511
			Rectangle	27.1 ^A^	1.3	4.8		29.3 ^A^	1.3	4.4	
			Line	28.3 ^A^	1.5	5.4		29.2 ^A^	1.5	5.2	
		Mean	Ellipse	29.9 ^A^	0.8	2.7	.022	30.1 ^A^	0.8	2.7	.024
			Rectangle	30.2 ^B^	0.7	2.4		30.2 ^A^	0.7	2.4	
			Line	29.5 ^A^	1.1	3.6		30.6 ^A^	1.1	3.5	
		Maximum	Ellipse	32.7 ^A^	1.0	3.0	**.000**	33.0 ^A^	1.0	2.9	.**000**
			Rectangle	32.9 ^A^	1.0	3.1		32.8 ^A^	1.0	3.1	
			Line	31.8 ^B^	0.7	2.1		32.6 ^B^	0.7	2.0	

Different letters indicate statistically significant differences between measured values

* Friedman test;

** Wilcoxon test

**Caption:** p = significance probability; ROI = region of interest; SD = standard deviation; VC = variation coefficient

Different letters indicate statistically significant differences between the measured values.

The comparison between nose and mouth temperatures during inspiration and expiration is shown in Table 3. The results indicate a statistically significant difference between all measurements, except for the mean inspiration temperature using the rectangle and the ellipse. The temperature of the mouth was always higher than that of the nose, both when inhaling and exhaling.

**Table 3:** Comparison of temperatures (°C) of the regions of interest between measures taken from the nose and mouth

Variables				Inhaling					Exhaling		
Temperature	**Breathing mode**	**Selection form**	**ROI**	**Mean**	**SD**	**VC**	**p-value**	**Mean**	**SD**	**VC**	**p-value**
Minimum	Nasal	Ellipse	Nose	22.0	1.3	6.1	**.000**	23.8	0.9	3.8	**.000**
			Mouth	27.3	1.4	5.0		29.1	1.1	3.9	
		Rectangle	Nose	22.0	1.3	6.0	**.000**	23.9	0.9	3.8	**.000**
			Mouth	27.2	1.5	5.4		29.3	0.9	3.1	
		Line	Nose	22.5	1.9	8.3	**.000**	24.5	0.8	3.3	**.000**
			Mouth	30.0	0.8	2.8		30.0	0.6	2.1	
	Mouth	Ellipse	Nose	22.0	1.2	5.3	**.000**	23.7	0.8	3.3	**.000**
			Mouth	27.3	1.3	4.8		29.1	1.1	3.8	
		Rectangle	Nose	22.0	1.2	5.3	**.000**	23.8	0.8	3.4	**.000**
			Mouth	27.2	1.3	4.8		29.3	1.0	3.2	
		Line	Nose	22.2	1.4	6.2	**.000**	24.2	0.8	3.2	**.000**
			Mouth	28.3	1.5	5.4		29.2	1.9	6.6	
Mean	Nasal	Ellipse	Nose	25.4	1.0	3.9	.819	26.5	0.7	2.6	**.000**
			Mouth	25.3	1.5	5.9		30.8	0.7	2.2	
		Rectangle	Nose	25.0	1.0	4.0	.169	26.4	0.7	2.7	**.000**
			Mouth	25.6	1.5	6.0		30.9	0.7	2.1	
		Line	Nose	24.1	1.2	5.2	**.000**	26.5	0.6	2.1	**.000**
			Mouth	30.9	1.0	3.1		30.9	0.6	2.1	
	Mouth	Ellipse	Nose	30.8	0.7	2.3	**.001**	26.2	0.7	2.6	**.000**
			Mouth	29.9	0.8	2.7		30.1	0.7	2.5	
		Rectangle	Nose	30.9	0.6	1.9	**.003**	26.0	0.7	2.6	**.000**
			Mouth	30.2	0.7	2.4		30.2	0.8	2.6	
		Line	Nose	24.5	1.1	4.5	**.000**	26.0	0.8	3.0	**.000**
			Mouth	29.5	1.1	3.6		30.6	1.5	5.0	
Maximum	Nasal	Ellipse	Nose	29.3	1.3	4.5	**.000**	29.5	0.7	2.4	**.000**
			Mouth	33.0	0.7	2.0		32.9	0.8	2.3	
		Rectangle	Nose	29.1	0.9	3.2	**.000**	29.6	0.7	2.3	**.000**
			Mouth	33.0	0.4	1.3		32.9	0.8	2.4	
		Line	Nose	25.7	1.2	4.8	**.000**	29.2	0.6	1.9	**.000**
			Mouth	32.3	1.1	3.5		32.4	1.0	3.0	
	Mouth	Ellipse	Nose	29.3	2.0	6.9	**.000**	29.0	1.1	3.9	**.000**
			Mouth	32.7	1.0	3.0		33.0	1.0	3.0	
		Rectangle	Nose	29.5	1.7	5.7	**.000**	29.1	1.0	3.3	**.000**
			Mouth	32.9	1.0	3.1		32.8	0.9	2.8	
		Line	Nose	26.2	1.3	5.0	**.000**	27.7	1.8	6.6	**.000**
			Mouth	31.8	0.7	2.1		32.6	0.8	2.5	

**Caption:** p = significance probability (Mann-Whitney test); ROI = Region of interest; DP = standard deviation; VC = variation coefficient

Lastly, the comparison between minimum, mean, and maximum temperatures (Table 4) showed a significant difference in all analyses, as expected. In general, mean temperatures had lower variation coefficients.

**Table 4:** Comparison of the images with the minimum, mean, and maximum temperatures

Variables				Inhaling				Exhaling			
ROI	**Breathing mode**	**Selection form**	**Temperature**	**Mean**	**SD**	**VC**	**p-value**	**Mean**	**SD**	**VC**	**p-value**
Nose	Nasal	Ellipse	Minimum	22.0 ^A^	1.3	6.1	**.000**	23.8 ^A^	0.9	3.8	**.000**
			Mean	25.4 ^B^	1.0	3.9		26.5 ^B^	0.7	2.6	
			Maximum	29.3 ^C^	1.3	4.5		29.5 ^C^	0.7	2.4	
		Rectangle	Minimum	22.0 ^A^	1.3	6.0	**.000**	23.9 ^A^	0.9	3.8	**.000**
			Mean	25.0 ^B^	1.0	4.0		26.4 ^B^	0.7	2.7	
			Maximum	29.1 ^C^	0.9	3.2		29.6 ^C^	0.7	2.3	
		Line	Minimum	22.5 ^A^	1.9	8.3	**.000**	24.5 ^A^	0.8	3.3	**.000**
			Mean	24.0 ^B^	1.2	5.2		26.5 ^B^	0.6	2.1	
			Maximum	25.7 ^C^	1.2	4.8		29.2 ^C^	0.6	1.9	
	Mouth	Ellipse	Minimum	22.0 ^A^	1.2	5.3	**.000**	23.7 ^A^	0.8	3.3	**.003**
			Mean	30.8 ^B^	0.7	2.3		26.2 ^B^	0.7	2.6	
			Maximum	29.2 ^C^	2.0	6.9		29.0 ^C^	1.1	3.9	
		Rectangle	Minimum	22.0 ^A^	1.2	5.3	**.000**	23.8 ^A^	0.8	3.4	**.000**
			Mean	30.9 ^B^	0.6	1.9		26.0 ^B^	0.7	2.6	
			Maximum	29.5 ^C^	1.7	5.7		29.0 ^C^	1.0	3.3	
		Line	Minimum	22.2 ^A^	1.4	6.2	**.000**	24.2 ^A^	0.8	3.2	**.000**
			Mean	24.5 ^B^	1.1	4.5		26.0 ^B^	0.8	3.0	
			Maximum	26.2 ^C^	1.3	5.0		27.7 ^C^	1.8	6.6	
Mouth	Nasal	Ellipse	Minimum	27.3 ^A^	1.4	5.0	**.000**	29.1 ^A^	1.1	3.9	**.000**
			Mean	25.3 ^B^	1.5	5.9		30.8 ^B^	0.7	2.2	
			Maximum	33.0 ^C^	0.7	2.0		32.9 ^C^	0.8	2.3	
		Rectangle	Minimum	27.2 ^A^	1.5	5.4	**.000**	29.3 ^A^	0.9	3.1	**.000**
			Mean	25.6 ^B^	1.5	6.0		30.9 ^B^	0.7	2.1	
			Maximum	32.9 ^C^	0.4	1.3		32.9 ^C^	0.8	2.4	
		Line	Minimum	30.0 ^A^	0.8	2.8	**.000**	30.0 ^A^	0.6	2.1	**.016**
			Mean	30.9 ^B^	1.0	3.1		30.9 ^B^	0.6	2.1	
			Maximum	32.3 ^C^	1.1	3.5		32.4 ^C^	1.0	3.0	
	Mouth	Ellipse	Minimum	27.3 ^A^	1.3	4.8	**.000**	29.0 ^A^	1.1	3.8	**.000**
			Mean	29.9 ^B^	0.8	2.7		30.1 ^B^	0.7	2.5	
			Maximum	32.7 ^C^	1.0	3.0		33.0 ^C^	1.0	3.0	
		Rectangle	Minimum	27.1 ^A^	1.3	4.8	**.000**	29.3 ^A^	1.0	3.2	**.000**
			Mean	30.2 ^B^	0.7	2.4		30.2 ^B^	0.8	2.6	
			Maximum	32.9 ^C^	1.0	3.1		32.8 ^C^	0.9	2.8	
		Line	Minimum	28.3 ^A^	1.5	5.4	**.000**	29.2 ^A^	1.9	6.6	**.000**
			Mean	29.5 ^B^	1.1	3.6		30.6 ^B^	1.5	5.0	
			Maximum	31.8 ^C^	0.7	2.1		32.6 ^C^	0.8	2.5	

Different letters indicate statistically significant differences between measured values

**Caption:** p = significance probability (Kruskal-Wallis test); ROI = region of interest; SD = standard deviation; VC = variation coefficient

## DISCUSSION

Despite being a preliminary study, the results obtained so far provide relevant evidence regarding the application and use of infrared thermography in objective breathing mode assessment, bringing important information about ROI selection and temperature analysis during inspiration and expiration.

The comparison of temperatures between the nasal and mouth/oronasal breathing modes showed that most of the differences were observed during inspiration when analyzing mean temperatures and that the mouth had more homogeneous data than the nose to assess and compare breathing modes. Thus, these proved to be important analysis parameters to distinguish breathing modes.

Three different forms - line, rectangle, and ellipse - in the FLIR Tools^®^ program were used to select the ROI temperatures (nose and mouth). There was a statistical difference between almost all measurements, predominantly between the line and ellipse and between the line and rectangle. There were no differences between the measurements of the rectangle and ellipse, showing that both selection forms extract temperatures similarly. The line obtained higher variation coefficients than the other forms in most measurements. This indicates greater data dispersion in relation to the mean when using this selection form, thus suggesting that the line may not obtain breathing temperatures as efficiently. It was also noted that during the manual selection of ROIs with the line, its position may vary from analysis to analysis, thus interfering with measurement repeatability. There were no differences between the measurements with the rectangle and ellipse, showing that both selection forms have similar temperature extraction results. The ellipse could be used instead of the rectangle because it comes closest to the anatomical shape of the mouth and nose and does not invade areas close to the ROI.

The comparison between nose and mouth temperatures shows that most mouth values are significantly higher than the nose ones. No studies were found in the literature that addressed this difference. Therefore, a protocol for evaluating breathing modes with infrared thermography must consider which region will be evaluated, whether the nose or the mouth. The comparison of temperature variation coefficients demonstrates that they tend to be lower in the mouth, especially during inspiration, thus indicating less data dispersion, which may point to more homogeneous results obtained in this region.

Statistically significant differences were found between all the mean, minimum, and maximum temperatures used for analysis - mean temperatures generally had lower variation coefficients. It was also found that, in general, the minimum and maximum temperatures were close when comparing those recorded in nasal and mouth/oronasal breathing, while the mean mouth temperatures using the rectangle and ellipse were able to distinguish the breathing mode, although only during inspiration. Therefore, mean temperatures can be considered the most suitable to assess breathing modes.

Despite the satisfactory results, this research has technical limitations due to its exploratory design. Among them are the small sample size and the fact that the assessments involved only nasal breathers without any respiratory complaints. The researchers aim to overcome these difficulties in future stages of the study, evaluating a larger sample, and including mouth/oronasal breathers in the trials.

The next phase of the research shall also have two raters analyzing the data to calculate interrater and intrarater agreement. The literature does not address this issue, but it seems essential for data reliability. Likewise, the normalized temperature should be calculated. Lastly, it should be noted that body movements during breathing can influence data collection. To minimize this impact, future research should use a method to monitor head movement during tests.

This research is innovative in oral motor therapy since there is no gold-standard equipment or technique for analyzing breathing modes. Furthermore, there is still little research involving the use of infrared thermography in breathing assessment, and the existing ones mainly analyze respiratory frequency to determine the number of respiratory cycles per minute^(8-19)^. A study aimed to analyze temperature modulation around both the nostrils and the mouth (as in the present study), but like other research, its main objective was to evaluate the respiratory rate by comparing it with shoulder movements during breathing^(18)^. Hence, no study has used this technique to verify breathing modes.

This tool is expected to be useful in speech-language-hearing clinical practice as a complementary analysis tool to detect mouth/oronasal breathing and provide simple and visual information to present the patient's progress throughout the therapeutic process.

## CONCLUSION

Breathing modes can be assessed with infrared thermography when analyzing the mean temperature of the mouth during inspiration using an ellipse.
