# Supplementary material for: Breathing mode assessment with thermography: a pilot study
Source: Codas. 2024 Apr 15;36(2):e20220323. doi: 10.1590/2317-1782/20232022323en (PMC11042686; doi:10.1590/2317-1782/20232022323en)
Supplement: Translation

# Translation: Avaliação do modo respiratório por meio da termografia: um estudo piloto

**DOI:** 10.1590/2317-1782/20232022323pt

**Authors:** Yasmim Carvalho Telson, Renata Maria Moreira Moraes Furlan, Rafael Augusto Magalhães Ferreira, Matheus Pereira Porto, Andréa Rodrigues Motta

**Affiliations:** 1 Programa de Pós-graduação em Ciências Fonoaudiológicas, Faculdade de Medicina, Universidade Federal de Minas Gerais - UFMG - Belo Horizonte (MG), Brasil.; 2 Departamento de Fonoaudiologia, Universidade Federal de Minas Gerais - UFMG - Belo Horizonte (MG), Brasil.; 3 Departamento de Engenharia Mecânica, Universidade Federal de Minas Gerais - UFMG - Belo Horizonte (MG), Brasil.

**Keywords:** Termografia, Fonoaudiologia, Respiração Bucal, Nariz, Boca

## Abstract

**Objetivo:**

Apresentar um método de análise do modo respiratório por meio da termografia infravermelha.

**Método:**

Estudo transversal exploratório de 38 imagens térmicas que representavam o momento da inspiração e da expiração durante a respiração nasal e durante a simulação da respiração oral de quatro voluntárias respiradoras nasais sem queixas respiratórias. Para a extração da temperatura das regiões de interesse (nariz e boca) foram utilizadas três formas de seleção distintas de dados (linha, retângulo e elipse) e três medidas de temperatura (mínima, média e máxima) por meio do software FLIR Tools®.

**Resultados:**

Dentre as três formas de seleção houve maior variabilidade nas medidas obtidas pela linha, revelando limitações nessa medida. Não houve diferenças entre as medidas do retângulo e elipse, mostrando que ambas as formas de seleção apresentam resultados semelhantes para a extração das temperaturas. Na comparação entre as temperaturas do nariz e da boca na inspiração e expiração, os resultados indicaram que houve diferença com relevância estatística em todas as medidas realizadas, exceto para as medidas de temperatura média da inspiração, usando o retângulo e a elipse. Percebe-se diferenciação do modo respiratório tanto na inspiração quanto na expiração quando utilizada a temperatura média da boca com o retângulo e a elipse.

**Conclusão:**

Sugere-se para avaliação do modo respiratório a avaliação da boca, por meio da elipse, com análise da temperatura média durante a inspiração.

## INTRODUÇÃO

A respiração é uma função fisiológica de grande relevância para a manutenção da vida^(1)^. O modo respiratório é um importante fator de influência no equilíbrio das estruturas e demais funções orofaciais, por isso demanda atenção por parte da Fonoaudiologia^(2)^. A respiração oral habitual pode causar alterações significativas no equilíbrio miofuncional, tais como: deformações craniofaciais e oclusais; perda de força da musculatura orofacial; alterações funcionais na mastigação, deglutição e fonoarticulação, além de modificações e adaptações corporais^(2)^.

Uma limitação da avaliação miofuncional é a escassez de técnicas quantitativas e precisas para a detecção de anormalidades funcionais e/ou estruturais que possam interferir no modo respiratório. A avaliação da respiração fica restrita a uma análise subjetiva do avaliador, já que não há um equipamento “padrão ouro” que possa ser utilizado^(3)^.

A termografia infravermelha consiste em um método sem contato que avalia um campo de temperaturas em uma determinada cena^(4)^. Trata-se de uma técnica não invasiva e não radioativa capaz de captar a faixa de radiação infravermelha emitida por um corpo ou um objeto e convertê-la em informação de temperatura^(5)^.

As câmeras termográficas ou termocâmeras são dispositivos compostos por sensores que respondem a uma faixa do espectro eletromagnético não percebida a olho nu (comprimento de onda entre 0,75 a 1000 μm)^(6)^. São responsáveis por transformar essas informações em sinais elétricos que são processados e convertidos em uma imagem visível, representada em diferentes escalas de cores para os vários níveis de temperatura captados^(4)^. De maneira geral, a radiação emitida pelo corpo não depende somente da sua temperatura, mas também da emissividade, da refletividade e da transmissividade^(7)^. Portanto a câmera termográfica registra de forma indireta a temperatura de um corpo, deduzindo-a por meio da quantidade de radiação que incide sobre suas lentes.

A termografia tem se mostrado uma importante ferramenta na investigação da função respiratória. Estudos promissores têm sido realizados baseando-se na premissa que a temperatura ao redor das narinas flutua no ciclo respiratório^(8-19)^. Durante a inspiração o ar frio do ambiente é inalado, já durante a expiração o ar quente dos pulmões é exalado^(9-11)^. Os termogramas são então, capazes de identificar tais mudanças e representar de forma qualitativa e quantitativa a variação de temperatura em torno do vestíbulo nasal.

O crescente interesse na termografia para avaliação da respiração decorre do fato de a frequência respiratória ser considerada um importante sinal vital^(9,10)^. Estudos também utilizam a técnica para detecção de apneia obstrutiva do sono durante a polissonografia^(12)^ e até mesmo para avaliar a respiração em casos de perfurações do septo nasal^(13)^. Por ser uma técnica que não exige contato, também tem sido utilizada na avaliação de crianças^(14)^ e de bebês em unidades neonatais^(15,16)^. Além de informação sobre a mudança de temperatura nas narinas, estudos utilizaram a termografia para analisar variações em torno da boca e obtiveram resultados robustos^(17-19)^.

Considerando a atual utilização da termografia como recurso de avaliação da função respiratória, foco de interesse do fonoaudiólogo no campo da Motricidade Orofacial, seria relevante integrar o uso desse instrumento na avaliação do modo respiratório. Assim, o presente estudo propõe apresentar um método de análise do modo respiratório por meio da termografia infravermelha.

## MÉTODO

Trata-se de um estudo transversal exploratório. A coleta dos dados só foi iniciada após aprovação pelo Comitê de Ética em Pesquisa da Instituição pelo seguinte número de parecer: 4.364.887. Todos os participantes assinaram o termo de consentimento livre e esclarecido.

Os critérios de inclusão para a seleção dos participantes foram: ter idade igual ou superior a 18 anos; ser respirador nasal; não apresentar alguma alteração física, neurológica e/ou cognitiva que impeça ou dificulte a colaboração durante a realização do exame. Já quanto aos critérios de exclusão foram: uso de bandagem sobre a pele e/ou outros fatores que impeçam sua exposição a um ambiente equilibrado de temperatura; realização de exercícios físicos, acupuntura, massagens ou ter sido submetido a estimulação elétrica, ter frequentado saunas ou ficado exposto de maneira prolongada ao sol até 24 horas antes da realização das medições; o uso de broncodilatadores e medicações vasoativas; presença de febre, rinite alérgica, sintomas gripais e/ou resfriado no dia dos testes; presença de dor orofacial; homens com presença de barba na face e mulheres no período fértil ou menstrual.

Por meio dos critérios de seleção descritos foram selecionadas por conveniência quatro voluntárias do sexo feminino, respiradoras nasais sem queixas respiratórias e com média de idade de 33,5 anos. As voluntárias eram fonoaudiólogas autodeclaradas respiradoras nasais atuantes na área de Motricidade Orofacial, sendo, portanto, confiável o modo respiratório nasal habitual relatado. No total, a amostra foi composta por 38 termogramas, sendo que 19 representavam a respiração nasal habitual e 19 a respiração oral/oronasal simulada por cada participante.

Ainda que apresentassem conhecimento sobre respiração oral, a classificação do modo respiratório das participantes foi confirmada em outras duas etapas. Assim, após o questionamento sobre o modo respiratório (podendo a resposta ser nasal, oral ou oronasal) as participantes foram submetidas a uma avaliação da possibilidade de vedamento labial por no mínimo 2 minutos. Para ser considerado possível respirador nasal a participante deveria ser capaz de manter o vedamento labial sem tensão. Por fim, foi realizada avaliação do tônus da musculatura orofacial, sendo necessário apresentar adequação para ser classificado como possível respirador nasal.

As participantes permaneceram no local cerca vinte minutos antes da medição para estabilização da temperatura, de acordo com as recomendações da Academia Americana de Termologia^(20)^.

Para realização dos ensaios foi utilizada uma câmera térmica FLIR SC660 *(FLIR Inc., Santa Barbara, CA)* e uma lente FLIR (38 mm) de 24 graus. A câmera FLIR SC660 foi selecionada por apresentar boa resolução (0,1 °C) e sensibilidade (0,03 °C). Foi escolhida a ferramenta de registro de sequência de imagens com uma frequência determinada, já que esta é capaz de gravar informações radiométricas (valores de temperatura). A frequência de aquisição escolhida foi de cinco frames por segundo. Já a seleção da lente, seguiu o critério de melhor aproveitamento do FOV (*Field of View*) para a cena, considerando uma distância de aproximadamente um metro entre a câmera e a face do voluntário, buscando-se assim maximizar o IFOV (*Instantaneous Field of View*) em relação aos alvos principais (nariz e boca). Desta forma, foi possível observar as mudanças de temperatura durante todo o processo de respiração.

As medições foram realizadas em uma sala com temperatura monitorada e mantida em torno de (20±1°C)^(20)^. Do ponto de vista metrológico, a temperatura do local deve ser bem controlada, para permitir a repetitividade dos resultados bem como criar uma referência térmica de base entre os diferentes voluntários^(4)^.

Quanto à montagem experimental dos testes, o termovisor foi estabilizado em um tripé para evitar vibrações durante os experimentos. Utilizou-se um ângulo de medição padrão de 30° para a melhor visualização das narinas e da boca durante a coleta. O participante foi então posicionado em uma cadeira fixa sem braços com encosto apoiado na parede, sentado e com os pés tocando o chão a cerca de um metro da lente da câmera.

A coleta dos dados foi dividida em duas etapas. Em um primeiro momento, foram realizadas filmagens com o voluntário respirando de forma habitual pelo nariz por dois minutos. Já na segunda etapa, os voluntários foram gravados, também por 2 minutos, simulando a respiração oral/oronasal. A Figura 1 representa a síntese desses processos.

**Figura 1:** Termogramas selecionados com o aparato experimental, durante o processo de inspiração e expiração pelo nariz (a)-(b), e pela boca (c)-(d)

Para análise quantitativa e qualitativa das sequências de imagens obtidas foi utilizado o *software* FLIR Tools^®^. O grau de emissividade escolhido foi de 0,98 que corresponde ao valor da pele humana^(21)^.

Para mensurar a variação de temperatura em torno das narinas e da boca durante a inspiração utilizaram-se três formas de seleção distintas: linha, elipse e retângulo. Primeiramente foram realizadas as medições posicionando-se uma linha horizontal entre as comissuras labiais e entre as narinas, depois os termogramas foram analisados usando a área de um retângulo e também de uma elipse posicionados entre as comissuras labiais, o arco do cupido e o limite inferior do lábio inferior e o mento para a obtenção dos valores térmicos da boca. Para obtenção da temperatura do nariz, outra elipse e outro retângulo foram posicionados circundando as narinas, o ápice do nariz e o ângulo nasolabial. As Figuras 2, 3, e 4 retratam os mecanismos utilizados.

**Figura 2:** Seleção manual dos ROIs no software Flir Tools por meio de um retângulo

**Figura 3:** Seleção manual das ROIs no software Flir Tools por meio de uma linha horizontal

**Figura 4:** Seleção manual das ROIs no software Flir Tools por meio de uma elipse

Ao selecionar a região de interesse (ROI) o programa Flir Tools^®^ apresenta três valores de temperatura sendo eles: o valor máximo, o valor mínimo e a média de temperatura da área selecionada.

Os dados foram analisados utilizando o programa de análise de dados IBM SPSS Statistics versão 23. Para testar a distribuição das variáveis contínuas aplicou-se o teste Shapiro-Wilk que indicou uma distribuição assimétrica. As análises comparativas entre o modo respiratório nasal e o modo respiratório oral/oronasal, bem como a comparação das temperaturas entre nariz e boca foram realizadas por meio do teste Mann-Whitney. A análise comparativa entre os valores mínimo, médio e máximo de temperatura por meio do teste Kruskal Wallis. O nível de significância adotado foi de 5%.

## RESULTADOS

Na Tabela 1 são comparados os valores de temperatura obtidos durante a respiração nasal e oral/oronasal, na inspiração e expiração, respectivamente. Percebe-se que as medidas com diferenças significantes na inspiração foram: temperatura média do nariz com a elipse e o retângulo; temperatura mínima da boca com a linha e a temperatura média da boca com a elipse, com o retângulo e com a linha. Já na expiração a medidas com diferenças estatísticas foram: temperatura média do nariz com a linha; temperatura máxima do nariz com a linha e temperatura média da boca com a elipse e o retângulo.

**Tabela 1:** Comparação dos valores das temperaturas (°C) das regiões de interesse durante a respiração nasal e oral/oronasal

**Variáveis**				**Inspiração**				**Expiração**			
**ROI**	**Temperatura Analisada**	**Forma de seleção**	**Modo Respiratório**	**Média**	**DP**	**CV**	**p valor**	**Média**	**DP**	**CV**	**p valor**
Nariz	Mínima	Elipse	RN	22,0	1,3	6,1	,990	23,8	0,9	3,8	,584
			RO	22,0	1,2	5,3		23,7	0,8	3,3	
		Retângulo	RN	22,0	1,3	6,0	,939	23,9	0,9	3,8	,750
			RO	22,0	1,2	5,3		23,8	0,8	3,4	
		Linha	RN	22,5	1,9	8,3	,603	24,5	0,8	3,3	,289
			RO	22,2	1,4	6,2		24,2	0,8	3,2	
	Média	Elipse	RN	25,4	1,0	3,9	**,000**	26,5	0,7	2,6	,197
			RO	30,8	0,7	2,3		26,2	0,7	2,6	
		Retângulo	RN	25,0	1,0	4,0	**,000**	26,4	0,7	2,7	,157
			RO	30,9	0,6	1,9		26,0	0,7	2,6	
		Linha	RN	24,1	1,2	5,2	,269	26,5	0,6	2,1	**,011**
			RO	24,5	1,1	4,5		26,0	0,8	3,0	
	Máxima	Elipse	RN	29,3	1,3	4,5	,932	29,5	0,7	2,4	,140
			RO	29,3	2,0	6,9		29,0	1,1	3,9	
		Retângulo	RN	29,1	0,9	3,2	,428	29,6	0,7	2,3	,076
			RO	29,5	1,7	5,7		29,1	1,0	3,3	
		Linha	RN	25,7	1,2	4,8	,324	29,2	0,6	1,9	**,001**
			RO	26,2	1,3	5,0		27,7	1,8	6,6	
Boca	Mínima	Elipse	RN	27,3	1,4	5,0	,962	29,1	1,1	3,9	,954
			RO	27,3	1,3	4,8		29,1	1,1	3,8	
		Retângulo	RN	27,2	1,5	5,4	,835	29,3	0,9	3,1	,930
			RO	27,2	1,3	4,8		29,3	1,0	3,2	
		Linha	RN	30,0	0,8	2,8	**,000**	30,0	0,6	2,1	,089
			RO	28,3	1,5	5,4		29,2	1,9	6,6	
	Média	Elipse	RN	25,3	1,5	5,9	**,000**	30,8	0,7	2,2	**,006**
			RO	29,9	0,8	2,7		30,1	0,7	2,5	
		Retângulo	RN	25,6	1,5	6,0	**,000**	30,9	0,7	2,1	**,003**
			RO	30,2	0,7	2,4		30,2	0,8	2,6	
		Linha	RN	30,9	1,0	3,1	**,000**	30,9	0,6	2,1	,363
			RO	29,5	1,1	3,6		30,6	1,5	5,0	
	Máxima	Elipse	RN	33,0	0,7	2,0	,224	32,9	0,8	2,3	,687
			RO	32,7	1,0	3,0		33,0	1,0	3,0	
		Retângulo	RN	33,0	0,4	1,3	,851	32,9	0,8	2,4	,821
			RO	32,9	1,0	3,1		32,8	0,9	2,8	
		Linha	RN	32,3	1,1	3,5	,124	32,4	1,0	3,0	,496
			RO	31,8	0,7	2,1		32,6	0,8	2,5	

**Legenda:** p = probabilidade de significância (Teste Mann-Whitney); RN = Respiração Nasal; RO = Respiração oral/oronasal; ROI = Região de Interesse; DP = desvio padrão; CV = coeficiente de variação

Na comparação das temperaturas de acordo com a forma de seleção tanto na inspiração quanto na expiração (Tabela 2) observou-se diferenças ao se comparar a temperatura entre linha, retângulo e elipse em quase todas as medidas. Essa diferença foi predominante entre a linha e elipse e linha e retângulo, sendo que na maioria das vezes as medidas da linha foram menores que as medidas do retângulo e da elipse.

**Tabela 2:** Comparação das medidas de temperaturas (°C) obtidas pelas diferentes formas de seleção: linha, elipse e retângulo

**Variáveis**				**Inspiração**				**Expiração**			
**ROI**	**Modo Respiratório**	**Temperatura**	**Forma de seleção**	Média**	**DP**	**CV**	Valor de p*	Média**	**DP**	**CV**	Valor de p*
Nariz	RN	Mínima	Elipse	22,0 ^A^	1,3	6,1	**,000**	23,8 ^A^	1,3	5,6	**,000**
			Retângulo	22,0^A^	1,3	6,0		23,9 ^A^	1,3	5,6	
			Linha	22,5 ^B^	1,9	8,3		24,5 ^B^	1,8	7,6	
		Média	Elipse	25,5 ^A^	1,0	3,9	**,000**	26,5 ^A^	1,0	3,8	**,037**
			Retângulo	25,0^B^	1,0	4,0		26,4 ^A^	1,0	3,8	
			Linha	24,0 ^C^	1,2	5,2		26,5 ^A^	1,2	4,7	
		Máxima	Elipse	29,3 ^A^	1,3	4,5	**,000**	29,5 ^A^	1,3	4,5	**,000**
			Retângulo	29,1 ^A^	0,9	3,2		29,6 ^A^	0,9	3,1	
			Linha	25,7 ^B^	1,2	4,8		29,2 ^B^	1,2	4,2	
	RO	Mínima	Elipse	22,0 ^A^	1,2	5,3	**,000**	23,7 ^A^	1,2	5,0	**,000**
			Retângulo	22,0 ^A^	1,2	5,3		23,8 ^A^	1,2	4,9	
			Linha	22,2 ^B^	1,4	6,2		24,2 ^B^	1,4	5,7	
		Média	Elipse	30,8 ^A^	0,7	2,3	**,000**	26,2 ^A^	0,7	2,7	**,**476
			Retângulo	30,9 ^A^	0,6	1,9		26,0 ^A^	0,6	2,3	
			Linha	24,5 ^B^	1,1	4,5		26,0 ^A^	1,1	4,2	
		Máxima	Elipse	29,3 ^A^	2,0	6,9	**,000**	29,0 ^A^	2,0	6,9	**,000**
			Retângulo	29,5 ^A^	1,7	5,7		29,0 ^A^	1,7	5,8	
			Linha	26,2 ^B^	1,3	5,0		27,7 ^B^	1,3	4,7	
Boca	RN	Mínima	Elipse	27,3 ^A^	1,4	5,0	**,000**	29,1 ^A^	1,4	4,7	**,026**
			Retângulo	27,2 ^A^	1,5	5,4		29,3 ^AB^	1,5	5,0	
			Linha	30,0 ^B^	0,8	2,8		30,0 ^C^	0,8	2,8	
		Média	Elipse	25,3 ^A^	1,5	5,9	**,000**	30,8 ^A^	1,5	4,8	**,568**
			Retângulo	25,6 ^A^	1,5	6,0		30,9 ^A^	1,5	5,0	
			Linha	30,9 ^B^	1,0	3,1		30,9 ^A^	1,0	3,1	
		Máxima	Elipse	33,0 ^A^	0,7	2,0	**,000**	32,9 ^A^	0,7	2,0	**,000**
			Retângulo	33,0 ^A^	0,4	1,3		32,9 ^A^	0,4	1,3	
			Linha	32,3 ^B^	1,1	3,5		32,4 ^B^	1,1	3,5	
	RO	Mínima	Elipse	27,3 ^A^	1,3	4,8	,191	29,0 ^A^	1,3	4,5	,511
			Retângulo	27,1 ^A^	1,3	4,8		29,3 ^A^	1,3	4,4	
			Linha	28,3 ^A^	1,5	5,4		29,2 ^A^	1,5	5,2	
		Média	Elipse	29,9 ^A^	0,8	2,7	,022	30,1 ^A^	0,8	2,7	,024
			Retângulo	30,2 ^B^	0,7	2,4		30,2 ^A^	0,7	2,4	
			Linha	29,5 ^A^	1,1	3,6		30,6 ^A^	1,1	3,5	
		Máxima	Elipse	32,7 ^A^	1,0	3,0	**,000**	33,0 ^A^	1,0	2,9	,**000**
			Retângulo	32,9 ^A^	1,0	3,1		32,8 ^A^	1,0	3,1	
			Linha	31,8 ^B^	0,7	2,1		32,6 ^B^	0,7	2,0	

Letras diferentes indicam diferenças estatisticamente significativas entre os valores mensurados

* teste de Friedman;

** Teste Wilcoxon

**Legenda:** p = probabilidade de significância; RN = Respiração nasal; RO = Respiração oral/oronasal; ROI = Região de interesse; DP = desvio padrão; DP= desvio padrão; CV = Coeficiente de variação

Já na tabela 3, são apresentadas a comparação das temperaturas mensuradas entre o nariz e a boca na inspiração e expiração. Os resultados indicam que houve diferença com relevância estatística em todas as medidas realizadas, exceto para as medidas de temperatura média da inspiração, usando o retângulo e a elipse. A temperatura da boca sempre se mostrou mais elevada do que a temperatura do nariz, tanto na inspiração quanto na expiração.

**Tabela 3:** Comparação das temperaturas (°C) das regiões de interesse entre as medidas realizadas no nariz e na boca

**Variáveis**				**Inspiração**					**Expiração**		
**Temperatura**	**Modo respiratório**	**Forma de seleção**	**ROI**	**Média**	**DP**	**CV**	**Valor de p**	**Média**	**DP**	**CV**	**Valor de p**
Mínima	RN	Elipse	Nariz	22,0	1,3	6,1	**,000**	23,8	0,9	3,8	**,000**
			Boca	27,3	1,4	5,0		29,1	1,1	3,9	
		Retângulo	Nariz	22,0	1,3	6,0	**,000**	23,9	0,9	3,8	**,000**
			Boca	27,2	1,5	5,4		29,3	0,9	3,1	
		Linha	Nariz	22,5	1,9	8,3	**,000**	24,5	0,8	3,3	**,000**
			Boca	30,0	0,8	2,8		30,0	0,6	2,1	
	RO	Elipse	Nariz	22,0	1,2	5,3	**,000**	23,7	0,8	3,3	**,000**
			Boca	27,3	1,3	4,8		29,1	1,1	3,8	
		Retângulo	Nariz	22,0	1,2	5,3	**,000**	23,8	0,8	3,4	**,000**
			Boca	27,2	1,3	4,8		29,3	1,0	3,2	
		Linha	Nariz	22,2	1,4	6,2	**,000**	24,2	0,8	3,2	**,000**
			Boca	28,3	1,5	5,4		29,2	1,9	6,6	
Média	RN	Elipse	Nariz	25,4	1,0	3,9	,819	26,5	0,7	2,6	**,000**
			Boca	25,3	1,5	5,9		30,8	0,7	2,2	
		Retângulo	Nariz	25,0	1,0	4,0	,169	26,4	0,7	2,7	**,000**
			Boca	25,6	1,5	6,0		30,9	0,7	2,1	
		Linha	Nariz	24,1	1,2	5,2	**,000**	26,5	0,6	2,1	**,000**
			Boca	30,9	1,0	3,1		30,9	0,6	2,1	
	RO	Elipse	Nariz	30,8	0,7	2,3	**,001**	26,2	0,7	2,6	**,000**
			Boca	29,9	0,8	2,7		30,1	0,7	2,5	
		Retângulo	Nariz	30,9	0,6	1,9	**,003**	26,0	0,7	2,6	**,000**
			Boca	30,2	0,7	2,4		30,2	0,8	2,6	
		Linha	Nariz	24,5	1,1	4,5	**,000**	26,0	0,8	3,0	**,000**
			Boca	29,5	1,1	3,6		30,6	1,5	5,0	
Máxima	RN	Elipse	Nariz	29,3	1,3	4,5	**,000**	29,5	0,7	2,4	**,000**
			Boca	33,0	0,7	2,0		32,9	0,8	2,3	
		Retângulo	Nariz	29,1	0,9	3,2	**,000**	29,6	0,7	2,3	**,000**
			Boca	33,0	0,4	1,3		32,9	0,8	2,4	
		Linha	Nariz	25,7	1,2	4,8	**,000**	29,2	0,6	1,9	**,000**
			Boca	32,3	1,1	3,5		32,4	1,0	3,0	
	RO	Elipse	Nariz	29,3	2,0	6,9	**,000**	29,0	1,1	3,9	**,000**
			Boca	32,7	1,0	3,0		33,0	1,0	3,0	
		Retângulo	Nariz	29,5	1,7	5,7	**,000**	29,1	1,0	3,3	**,000**
			Boca	32,9	1,0	3,1		32,8	0,9	2,8	
		Linha	Nariz	26,2	1,3	5,0	**,000**	27,7	1,8	6,6	**,000**
			Boca	31,8	0,7	2,1		32,6	0,8	2,5	

**Legenda:** p = probabilidade de significância (Teste Mann- Whitney); RN = Respiração Nasal; RO = Respiração oral/oronasal; ROI = Região de Interesse; DP = desvio padrão; CV = Coeficiente de variação

Por fim, na comparação das temperaturas mínima, média e máxima (tabela 4) verificou-se, como esperado, que existe diferença significante em todas as análises. De maneira geral, as temperaturas médias apresentaram menores coeficientes de variação.

**Tabela 4:** Comparação das imagens das medidas realizadas com a temperatura mínima, média e máxima

**Variáveis**				**Inspiração**				**Expiração**			
**ROI**	**Modo respiratório**	**Forma de seleção**	**Temperatura**	**Média**	**DP**	**CV**	**Valor de p**	**Média**	**DP**	**CV**	**Valor de p**
Nariz	RN	Elipse	Mínima	22,0 ^A^	1,3	6,1	**,000**	23,8 ^A^	0,9	3,8	**,000**
			Média	25,4 ^B^	1,0	3,9		26,5 ^B^	0,7	2,6	
			Máxima	29,3 ^C^	1,3	4,5		29,5 ^C^	0,7	2,4	
		Retângulo	Mínima	22,0 ^A^	1,3	6,0	**,000**	23,9 ^A^	0,9	3,8	**,000**
			Média	25,0 ^B^	1,0	4,0		26,4 ^B^	0,7	2,7	
			Máxima	29,1 ^C^	0,9	3,2		29,6 ^C^	0,7	2,3	
		Linha	Mínima	22,5 ^A^	1,9	8,3	**,000**	24,5 ^A^	0,8	3,3	**,000**
			Média	24,0 ^B^	1,2	5,2		26,5 ^B^	0,6	2,1	
			Máxima	25,7 ^C^	1,2	4,8		29,2 ^C^	0,6	1,9	
	RO	Elipse	Mínima	22,0 ^A^	1,2	5,3	**,000**	23,7 ^A^	0,8	3,3	**,003**
			Média	30,8 ^B^	0,7	2,3		26,2 ^B^	0,7	2,6	
			Máxima	29,2 ^C^	2,0	6,9		29,0 ^C^	1,1	3,9	
		Retângulo	Mínima	22,0 ^A^	1,2	5,3	**,000**	23,8 ^A^	0,8	3,4	**,000**
			Média	30,9 ^B^	0,6	1,9		26,0 ^B^	0,7	2,6	
			Máxima	29,5 ^C^	1,7	5,7		29,0 ^C^	1,0	3,3	
		Linha	Mínima	22,2 ^A^	1,4	6,2	**,000**	24,2 ^A^	0,8	3,2	**,000**
			Média	24,5 ^B^	1,1	4,5		26,0 ^B^	0,8	3,0	
			Máxima	26,2 ^C^	1,3	5,0		27,7 ^C^	1,8	6,6	
Boca	RN	Elipse	Mínimo	27,3 ^A^	1,4	5,0	**,000**	29,1 ^A^	1,1	3,9	**,000**
			Média	25,3 ^B^	1,5	5,9		30,8 ^B^	0,7	2,2	
			Máxima	33,0 ^C^	0,7	2,0		32,9 ^C^	0,8	2,3	
		Retângulo	Mínima	27,2 ^A^	1,5	5,4	**,000**	29,3 ^A^	0,9	3,1	**,000**
			Média	25,6 ^B^	1,5	6,0		30,9 ^B^	0,7	2,1	
			Máxima	32,9 ^C^	0,4	1,3		32,9 ^C^	0,8	2,4	
		Linha	Mínima	30,0 ^A^	0,8	2,8	**,000**	30,0 ^A^	0,6	2,1	**,016**
			Média	30,9 ^B^	1,0	3,1		30,9 ^B^	0,6	2,1	
			Máxima	32,3 ^C^	1,1	3,5		32,4 ^C^	1,0	3,0	
	RO	Elipse	Mínima	27,3 ^A^	1,3	4,8	**,000**	29,0 ^A^	1,1	3,8	**,000**
			Média	29,9 ^B^	0,8	2,7		30,1 ^B^	0,7	2,5	
			Máxima	32,7 ^C^	1,0	3,0		33,0 ^C^	1,0	3,0	
		Retângulo	Mínima	27,1 ^A^	1,3	4,8	**,000**	29,3 ^A^	1,0	3,2	**,000**
			Média	30,2 ^B^	0,7	2,4		30,2 ^B^	0,8	2,6	
			Máxima	32,9 ^C^	1,0	3,1		32,8 ^C^	0,9	2,8	
		Linha	Mínima	28,3 ^A^	1,5	5,4	**,000**	29,2 ^A^	1,9	6,6	**,000**
			Média	29,5 ^B^	1,1	3,6		30,6 ^B^	1,5	5,0	
			Máxima	31,8 ^C^	0,7	2,1		32,6 ^C^	0,8	2,5	

Letras diferentes indicam diferenças estatisticamente significativas entre os valores mensurados

**Legenda:** p = probabilidade de significância (Teste Kruskal Wallis); RN = Respiração nasal; RO = Respiração oral/oronasal; ROI = Região de interesse; DP = Desvio Padrão; CV = Coeficiente de variação

## DISCUSSÃO

Apesar de ser um estudo preliminar, os resultados obtidos até então fornecem evidências relevantes quanto à aplicação e uso da termografia infravermelha na avaliação objetiva do modo respiratório trazendo informações importantes quanto a seleção das regiões de interesse e análise das temperaturas durante a inspiração e expiração.

Na comparação das temperaturas entre os modos respiratórios nasal e oral/oronasal observou-se que a maior parte das diferenças foi observada na inspiração ao se analisar a temperatura média e que a seleção da boca para avaliação e comparação do modo respiratório mostrou dados mais homogêneos em relação à utilização do nariz. Assim, esses se mostraram parâmetros importantes de análise para diferenciação do modo respiratório.

Sobre as ferramentas para seleção das temperaturas das regiões de interesse (nariz e boca) foram utilizadas três formas distintas existentes no programa FLIR Tools^®^: linha, retângulo e elipse. Houve diferença estatística ao se comparar praticamente todas as medidas entre si. Percebe-se que essa diferença foi predominante entre “linha e elipse” e “linha e retângulo”. Não houve diferenças entre as medidas do retângulo e elipse, mostrando que ambas as formas de seleção apresentam resultados semelhantes para a extração das temperaturas. Na maioria das mensurações os coeficientes de variação obtidos pela forma de seleção “linha” foram maiores, em comparação às demais medidas. Tal fato indica que houve uma maior dispersão dos dados em relação à média quando utilizada esta forma de seleção, sugerindo assim, que a linha pode não ser tão eficiente para a obtenção das temperaturas durante a respiração. Percebe-se também que durante a seleção manual das regiões de interesse quando utilizada a linha, sua posição pode variar de análise para análise interferindo assim, na repetitividade das medições. Não houve diferenças entre as medidas do retângulo e elipse, mostrando que ambas as formas de seleção apresentam resultados semelhantes para a extração das temperaturas. A elipse poderia ser utilizada em detrimento do retângulo por ser a que mais se aproximou da forma anatômica da boca e do nariz e não invadiu áreas próximas à região de interesse.

Quanto às medidas realizadas comparando as temperaturas do nariz e da boca, percebe-se que a maioria dos valores da boca são significativamente maiores que os do nariz. Não foram encontrados estudos na literatura que abordassem essa diferença. Dessa forma, um protocolo para avaliação do modo respiratório por meio da termografia infravermelha deve levar em conta qual região será avaliada: nariz ou boca. Comparando os coeficientes de variação das temperaturas obtidas, percebe-se que esses tendem a serem menores para a boca, especialmente na inspiração, indicando assim, menor dispersão dos dados, o que pode indicar resultados mais homogêneos obtidos para essa região.

Em relação às temperaturas utilizadas para análise (média, mínima ou máxima) foram encontradas diferenças estatisticamente significantes entre todas as medidas comparadas, sendo que as temperaturas médias foram as que apresentaram, de maneira geral, valores menores de coeficiente de variação. Verificou-se ainda que, de maneira geral, as temperaturas mínima e máxima foram próximas ao se comparar a medidas registradas na respiração nasal e oral/oronasal, já as medidas de temperatura média da boca usando as formas de seleção retângulo e elipse conseguiram realizar a diferenciação do modo respiratório, embora apenas na inspiração. Assim, pode-se considerar as temperaturas médias como as mais indicadas para avaliação do modo respiratório.

Apesar dos resultados satisfatórios encontrados, a pesquisa desenvolvida apresenta limitações técnicas devido seu caráter exploratório. Dentre eles está o tamanho amostral reduzido e o fato de as avaliações terem ocorrido somente com indivíduos respiradores nasais sem nenhuma queixa respiratória. Pretende-se contornar essas dificuldades em etapas posteriores do estudo em que serão avaliados um número maior de participantes, bem como serão incluídos respiradores orais/oronasais nos ensaios.

Outro aspecto que deve ser observado na próxima fase da pesquisa é que a análise dos dados seja realizada por dois avaliadores distintos para cálculo da concordância inter e intra avaliador. Esse não é um aspecto levantado na literatura, mas parece fundamental para a confiabilidade dos dados. Também se sugere que seja realizado o cálculo da temperatura normalizada. Por fim, ressalta-se também que os movimentos do corpo durante a respiração podem influenciar na coleta dos dados, para minimizar este impacto, sugere-se a utilização de um método para monitoramento do movimento da cabeça durante os testes.

Esta pesquisa é inovadora dentro da área da Motricidade Orofacial uma vez que não existe um equipamento ou técnica “padrão ouro” para análise do modo respiratório. Além disso, ainda há poucas pesquisas envolvendo a utilização da termografia infravermelha na avaliação da respiração e estas têm como objetivo principal a análise da frequência respiratória, isto é, a determinação do número de ciclos respiratórios por minuto^(8-19)^. Um estudo se propôs analisar não somente a modulação de temperatura em torno das narinas, mas também em torno da boca como fizemos, mas assim como as demais pesquisas seu objetivo principal foi avaliar a taxa respiratória comparando-a com os movimentos dos ombros durante a respiração^(18).^ Portanto, não há estudos utilizando essa técnica para a verificação do modo respiratório.

Espera-se que esta ferramenta possa ser empregada na prática clínica fonoaudiológica como uma ferramenta complementar de análise para detecção da respiração oral/oronasal, bem como também possa ser utilizada no tratamento como uma fonte de informação simples e visual para apresentar a evolução do paciente ao longo do processo terapêutico.

## CONCLUSÃO

O modo respiratório pode ser avaliado por meio da termografia infravermelha quando se analisa a temperatura média da boca durante a inspiração por meio de uma elipse.
